# A novel auxiliary fixation technique of meshes in intraperitoneal onlay mesh procedures for incisional hernia repair

**DOI:** 10.3389/fsurg.2023.1201992

**Published:** 2023-06-20

**Authors:** Kunjie Zhang, Jiayu Lin, Lianjin Qin, Leqi Li, Changjun Xia, Jianfang Li

**Affiliations:** ^1^ Department of General Surgery, The First Affiliated Hospital of Henan University of Traditional Chinese Medicine, Zhengzhou, China; ^2^ Department of Obstetrics and Gynaecology, Li Ka Shing Faculty of Medicine, The University of Hong Kong, Kong Kong S.A.R., China; ^3^ Shenzhen Key Laboratory of Fertility Regulation, Reproductive Medicine Center, The University of Hong Kong-Shenzhen Hospital, Shenzhen, China; ^4^ Department of Ventral Hernia Surgery, The First People’s Hospital of Huzhou, Huzhou, China; ^5^ Department of General Surgery, Henan Hongli Hospital, Xinxiang, China; ^6^ Department of Hernia and Abdominal Wall Surgery, The First People's Hospital of Xiaoshan District in Hangzhou, Hangzhou, China

**Keywords:** magnet attraction, incisional hernia, intraperitoneal onlay mesh, laparoscopy, fixation

## Abstract

**Introduction:**

Mesh fixation is an important step in incisional hernia repair. Weak fixation possibly results in postoperative pain, and even hernia recurrence. We innovated an auxiliary fixation approach, the magnet attraction technique (MAT), to achieve better mesh fixation. The purpose of this study was to evaluate the effect of MAT in intraperitoneal onlay mesh (IPOM) procedures for incisional hernia repair.

**Methods:**

Historical patient records were analyzed according to the clinical data of 16 patients with incisional hernias. Among them, 5 patients have undergone IPOM repair procedures in combination with MAT to assist in mesh fixation. As a control, 11 patients treated with IPOM and mesh fixation via conventional suspension were included. The clinical data collected include patients' basic characteristics, intraoperative and postoperative conditions, and follow-up results in both groups.

**Results:**

Compared with patients in the control group, patients in the MAT group were found to suffer from a larger hernia ring diameter and longer surgical duration, but shorter hospitalization length on average. And most importantly, no complication has been reported in the MAT group.

**Conclusion:**

MAT in IPOM operation was regarded as a feasible and safe technique for patients suffering from incisional hernias.

## Introduction

Incisional hernia (IH) is considered to be one of the most frequent complications of abdominal surgery, with a cumulative incidence rising to 30% (1, 2). Age and infection have been reported as independent risk factors for IH, and in the meanwhile, obesity contributes to the development of complex IH (3, 4).

As IH is not self-limiting, prompt surgical treatment is of great importance for its harmful impact on patients' future life quality, even in its early stage (5). And hernia repair has grown into one of the most commonly performed procedures in general surgeries (6).

For patients without surgical contraindications, sufficient perioperative preparation, and active surgical treatment is of significant need. With the continuous optimization of surgical approaches, a variety of new repair surgeries have been innovated, which can be open, laparoscopic, or hybrid. And these years, the advances of robot-assisted operations in IH repair have also gained great popularity (6). IPOM is a laparoscopic procedure that is adapted mostly (7). It is preferred for its convenience to operate and no need to separate the tissue of the abdominal wall. In IPOM, the accurate placement of meshes is an important step, which requires the alignment of the mesh's center to the center of the hernia ring to prevent further recurrence. Nevertheless, due to the difficulty in extending the mesh fully in the intraperitoneal, satisfying alignment and mesh fixation can hardly be achieved.

Based on the above clinical experience, we innovated MAT in IPOM operation for good fixation of the meshes. And by analyzing our historical clinical data, our study aimed to investigate the effectiveness of MAT in IPOM during IH repair.

## Materials and methods

### General information

We analyzed the clinical data from sixteen patients having suffered from IH. And they were admitted to the Department of General Surgery, the First Affiliated Hospital of Henan University of Chinese Medicine and The Department of Ventral Hernia Surgery, The First People's Hospital of Huzhou from March 2019 to February 2023. All of them were treated with IPOM, among whom 5 patients were operated on with MAT in the fixation of meshes and 11 patients with conventional suspension. Patients' characteristics, intraoperative conditions, as well as postoperative complications, were recorded by clinical observation and postoperative follow-ups.

### Surgical procedure

General anesthesia was performed by endotracheal intubation. A 10 mm operative hole was established firstly for the laparoscope to get access to. Afterwards, 2 other operative holes at the diameter of 5 mm and 12 mm were created for the later entry of an atraumatic forceps and meshes. When the hernia ring was found, the corresponding position of its center would be marked on the ventral wall. After the adhesion around the hernia defect was fully separated, 2–0 non-absorbable sutures were utilized to close the hernia ring.

**Figure 1:**
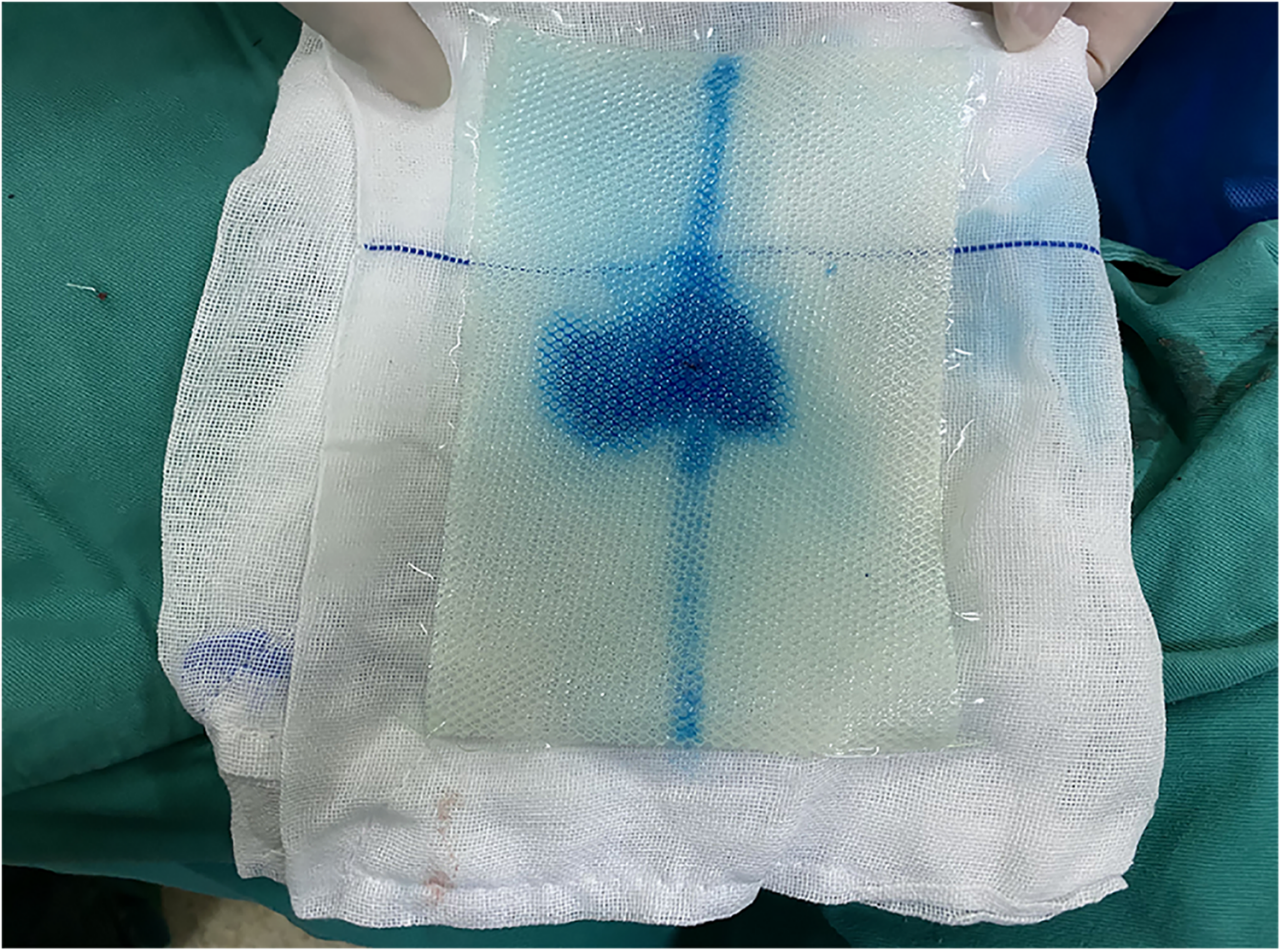
The center of the mesh was marked with methylene blue.

**Figure 2:**
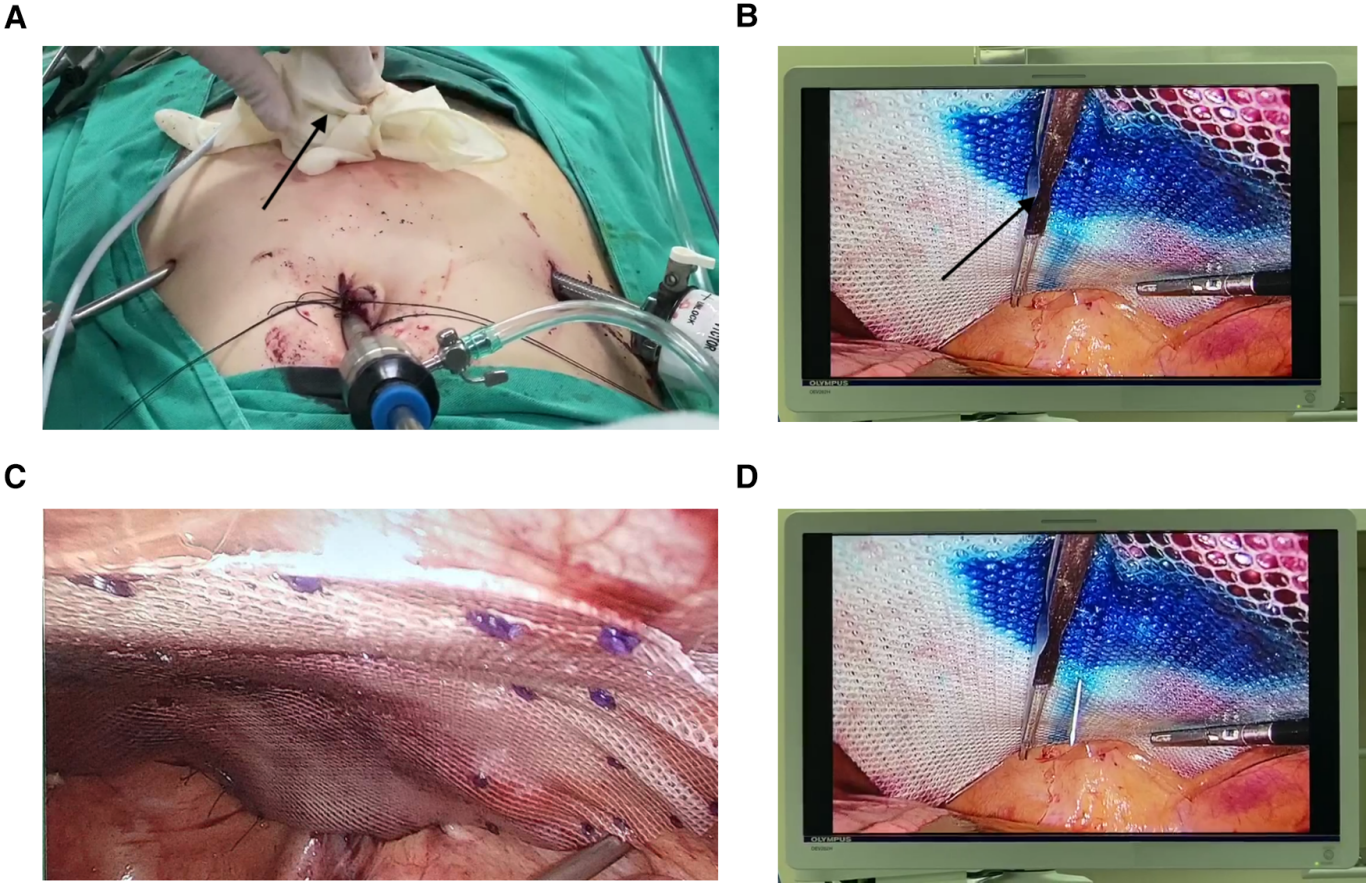
The components of the MAT technique. (**A**) A glove-wrapped magnet (the black arrow) was placed on the abdominal wall. (**B**) On the corresponding site to the magnet in the ventral cavity, a scalpel handle (the black arrow) was placed to help stretch the mesh. (**C**) The fully stretched mesh. (**D**) A needle would be utilized to measure the alignment of the hernia defect center and the mesh's center.

Following that, in the MAT group, the mesh's center was marked with methylene blue firstly to make it more visible (Figure 1). The mesh and the scalpel handle were closely kept on the dorsal face of the abdominal wall, while the magnet inside a sterile glove would be placed on the corresponding site of the hernia ring's center on the surface of the ventral wall indicated above (Figures 2A, B). The mesh would thereby be adhered to the abdominal wall and kept stretched through the magnetic pull between the scalpel handle and magnet (Figure 2C). Secondly, a 10 ml syringe needle would be inserted vertically through the projection site on the abdominal wall till the mesh (Figure 2D). If the needle went right through the mesh's center, it suggested that the center of the mesh was aligned with the center of the hernia ring. If not, a withdrawal of the needle to the muscle or the subcutaneous fat layer would be needed. Then, a slight adjustment of the mesh's position would be performed by moving the magnet on the ventral surface and the scalpel handle by an atraumatic forceps. The needle would be re-inserted to verify the alignment of the 2 centers. When a satisfactory alignment was achieved, the mesh's fixation would be carried out by an atraumatic forceps in one hand to stretch the mesh further and a nail gun in the other hand to fix the mesh.

In the control group, the conventional suspension approach was applied in the mesh fixation. Briefly, sewing 4 threads through the anti-adhesion mesh was first performed followed by knotting. And a portion of the threads' length was left aside. The meshes were then placed into the abdominal cavity along with the threads. Suture passers were thereafter utilized to puncture into the ventral cavity and pull the threads out of the abdomen. Meshes got stretched accordingly. However, if the meshes were examined not to cover the hernia ring properly, repiercing and repositioning would be performed until a satisfying coverage was achieved. Knotting was finally executed in the subcutaneous layer to fix the meshes.

After the fixation, a drainage tube would be placed between the bowel and the mesh for adequate drainage. Finally, the ventral wall got sutured.

### Postoperative management and follow-up

During one month after the surgery, patients' abdomens would be continuously immobilized with an abdominal bandage to prevent bleeding, as well as promote the healing of the hernia defect. The drainage tubes would not be removed until a patient's drainage volume was less than 10 ml in one day. All patients were followed up by telephone and outpatient re-examination.

### Statistical analysis

All quantitative data were represented by mean ± standard error of measurement. Chi-square test was used to compare qualitative data. And the statistical analysis was performed by the software of SPSS 25.0 (IBM Corp., Armonk, NY, USA).

## Results

The basic characteristics of the patients were summarized in Table 1. In terms of age, BMI, or gender composition, there were no statistically significant differences between the two groups.

**Table 1:** Patients’ characteristics.

	Total (*n* = 16)	MAT (*n* = 5)	Conventional Suspension (*n* = 11)	*P* value
Male (sex)	6	3	3	0.210
Age (years)	63.31 ± 3.30	52.80 ± 6.58	68.09 ± 2.92	0.081
BMI (kg/m^2^)	24.31 ± 0.98	25.80 ± 1.49	23.63 ± 1.25	0.323

Intraoperative and postoperative data were presented in Table 2. According to the analysis, overal, MAT patients endured a greater hernia ring diameter and longer operation time, but a shorter length of stay on average, although they were not significantly different. During follow-up at least 3 months after surgery, the patients in the MAT group experienced no complications, such as recurrence, seroma and infection, while chronic pain was reported in 3 patients and a local recurrence was reported in 1 patient in the conventional suspension group.

**Table 2:** Intraoperative and postoperative parameters.

	Total (*n* = 16)	MAT (*n* = 5)	Conventional suspension (*n* = 11)	*P* value
The diameter of the hernia defect (cm)	7.13 ± 0.93	9.80 ± 1.59	5.91 ± 0.96	0.075
Operation time (minutes)	190.00 ± 14.85	226.00 ± 20.88	173.64 ± 17.77	0.103
Duration of hospitalization (days)	9.31 ± 1.07	7.80 ± 1.24	10.00 ± 1.43	0.357
Complications reported	4	0	4	0.119

## Discussion

In IPOM procedures, good fixation of the meshes is an important prerequisite to reduce future recurrence for patients with IH. According to our clinical practice, the mesh was liable to curl and could not be evenly paved in the abdominal cavity during the fixation, which would weaken its function of preventing the abdominal contents from extruding out. It can be foreseen that some long-term complications, such as recurrence, would thereafter be caused. Thus, we innovated an auxiliary technique named MAT to help stretch the mesh for better fixation. That is, the scalpel handle in the abdominal cavity will be attracted by the external magnet placed on the abdominal wall, and the mesh between them will get a full extension by means of the attraction.

The technique of MAT has its advantages. Firstly, the mesh can get adequately stretched by the attraction between the scalpel handle in the abdominal cavity and the magnet on the ventral wall. Secondly, this attraction with moderate strength will allow the mesh to move on a fairly large scale until it reaches its exact position, which is conducive to fixation. Finally, it helps to reduce the surgeon's workload and operation time, as merely one, rather than two atraumatic forceps as the full-thickness mesh fixation has been done (8), will be required to further adjust the mesh's position. For the surgeon, it's feasible to keep an atraumatic forceps in one hand and operate a nail gun to complete the final fixation of the mesh in the other hand.

At present, as the control group in our study was treated, most clinical procedures use the traditional approach of suspension to stretch the meshes (8). Meshes get stretched mainly by pulling the threads outside the abdominal cavity. However, some disadvantages exist in this technique. For one thing, at least four puncture sites need to be pierced with suture passers, which will potentially aggravate patients' postoperative pain. For another, this technique will not permit frequent repositionings of the mesh if it is found improperly covering the hernia defect.

**Figure 3:**
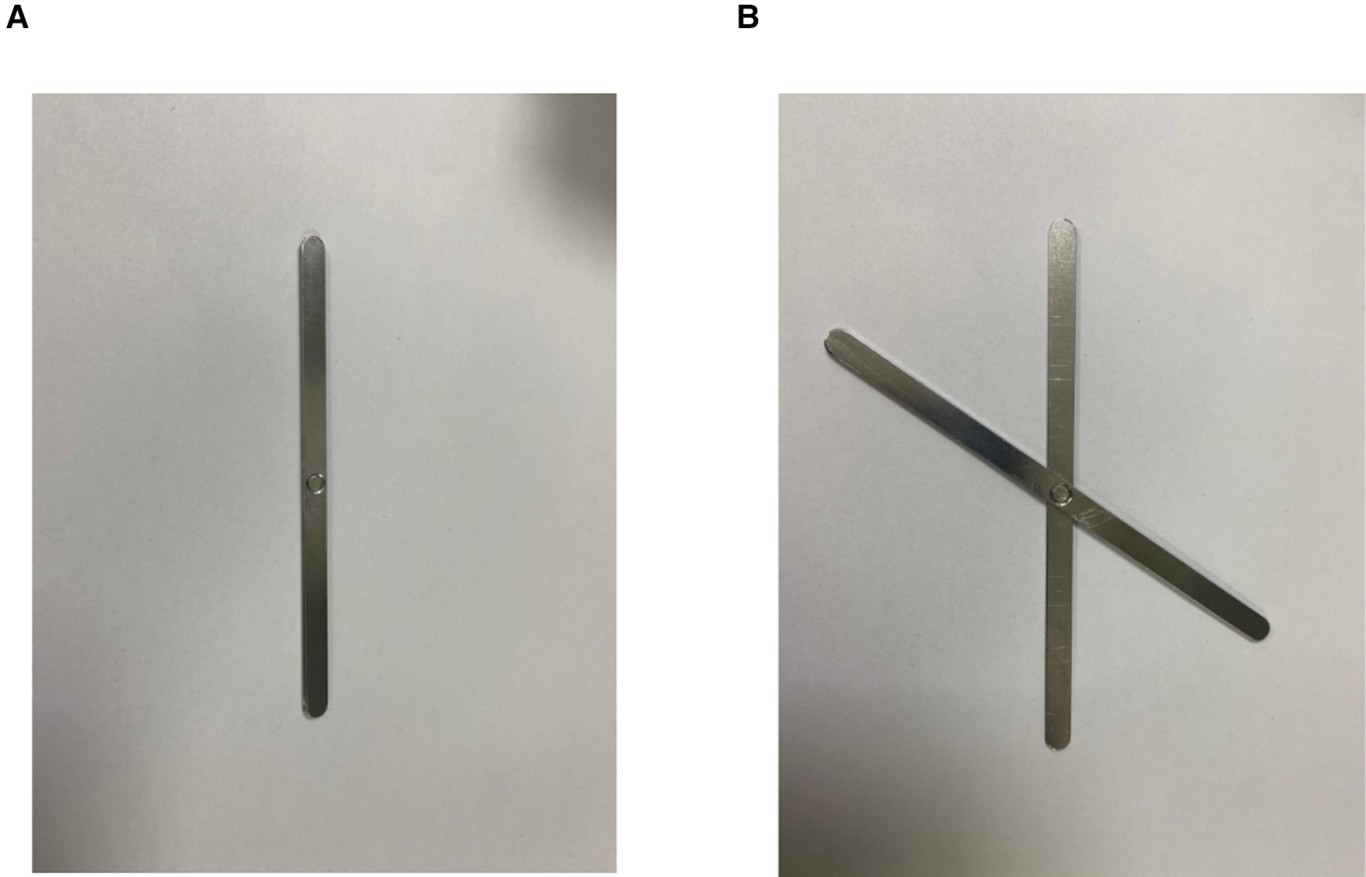
A better device to replace the scalpel handle. (**A**) The device was in a folded state. (**B**) The device was in an open state.

Similar to the scalpel handle, a metal clip can also be considered an auxiliary fixation device in MAT. Among the five patients using MAT in IPOM, scalpel handles were used in 4 patients and a metal clip was used in one. At present, we have developed a better auxiliary device to replace the scalpel handle. Due to its larger surface area after unfolding (Figure 3), it will be more effective in attracting the mesh to the ventral wall.

Although the MAT technique has only been applied in patients with IH, it can be speculated that MAT can also be applied to those patients suffering from umbilical hernias and linea alba hernias.

## Limitations

There are several limitations in our study, too. First of all, it is an analysis with a small sample size. More cases need to be collected and analyzed. Secondly, due to its retrospective nature, recall bias may exist in the follow-up.

## Conclusions

In conclusion, MAT is a feasible and safe technique for mesh fixation during incisional hernia repair in IPOM procedures. Like the conventional suspension approach, it can help stretch the mesh. Given the mobility of the magnet, the mesh's repositioning process gets simplified without several punctures. Benefiting from it, postoperative complications, including long-term recurrence, will be hopefully reduced in MAT patients. More cases and clinical experience are expected to be incorporated to verify the durability of the MAT in IPOM for hernia repair.

## Data Availability

The original contributions presented in the study are included in the article/Supplementary Material, further inquiries can be directed to the corresponding author/s
